# Supplementary material for: The Glucosylceramide Synthase Inhibitor PDMP Causes Lysosomal Lipid Accumulation and mTOR Inactivation
Source: Int J Mol Sci. 2021 Jun 30;22(13):7065. doi: 10.3390/ijms22137065 (PMC8268262; doi:10.3390/ijms22137065)
Supplement: Supplementary file 1 [file ijms-22-07065-s001.zip › ijms-1232646-supplementary.pdf]

Supplementary Materials

Figure S1

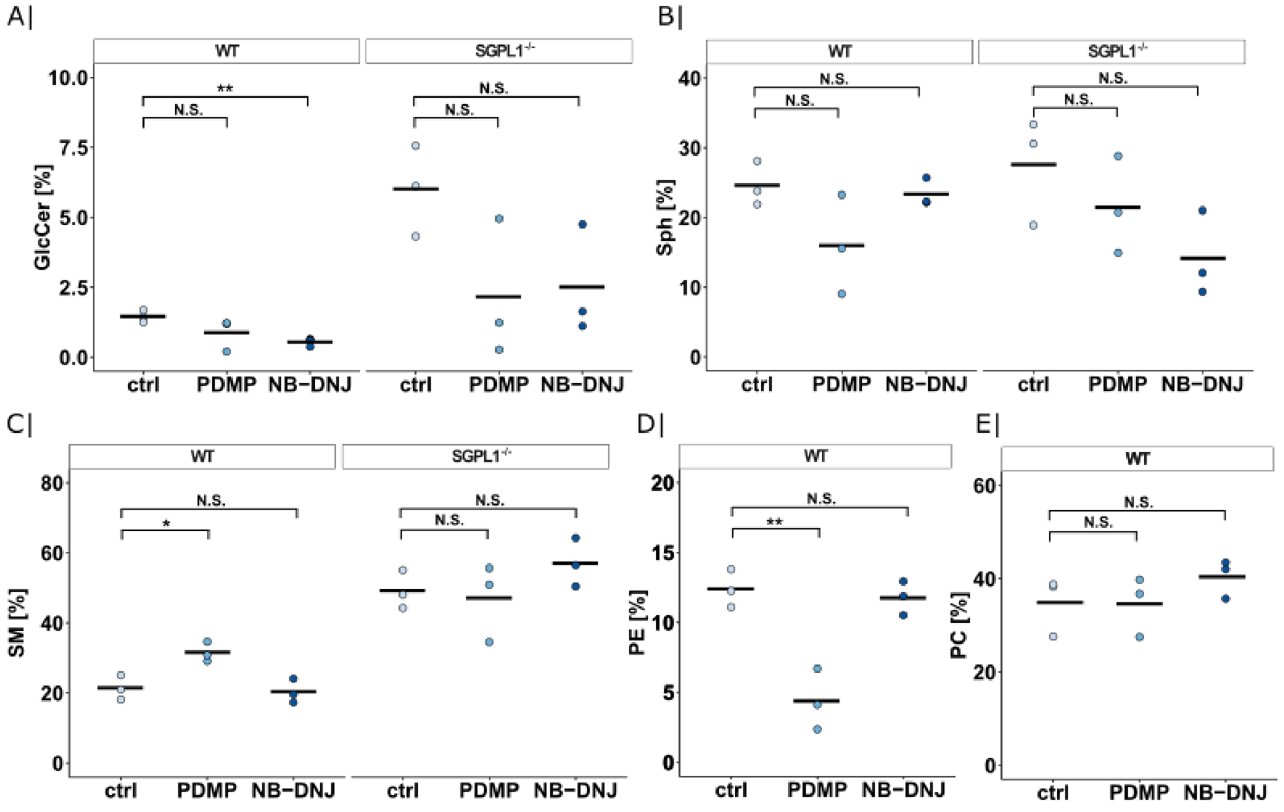

Figure S1: Quantification of A| GlcCer, B| Sph C| SM, D| PE and E| PC levels in Fig. 1b. The fluorescent signal corresponding to the respective band was divided by the total intensity of all fluorescently labelled lipids. This was extracted from 3 independent experiments and is presented as dot plot including the calculated mean. Welch two sample t-tests were performed between control and PDMP or NB-DNJ conditions (A| WT ctrl-PDMP: N.S. $P = 2.2\times10^{-1}$, A| WT ctrl-NB-DNJ: ** $P = 5.4\times10^{-3}$, A| SGPL1$^{-/-}$ ctrl-PDMP: N.S. $P = 9.8\times10^{-2}$, A| SGPL1$^{-/-}$ ctrl-NB-DNJ: N.S. $P = 7.9\times10^{-2}$, B| WT ctrl-PDMP: N.S. $P = 1.6\times10^{-1}$, B| WT ctrl-NB-DNJ: N.S. $P = 6.1\times10^{-1}$, B| SGPL1$^{-/-}$ ctrl-PDMP: N.S. $P = 3.6\times10^{-1}$, B| SGPL1$^{-/-}$ ctrl-NB-DNJ: N.S. $P = 7.9\times10^{-2}$, C| WT ctrl-PDMP: * $P = 1.9\times10^{-2}$, C| WT ctrl-NB-DNJ: N.S. $P = 7.2\times10^{-1}$, C| SGPL1$^{-/-}$ ctrl-PDMP: N.S. $P = 7.9\times10^{-1}$, C| SGPL1$^{-/-}$ ctrl-NB-DNJ: N.S. $P = 1.9\times10^{-1}$, D| WT ctrl-PDMP: ** $P = 9.3\times10^{-3}$, D| WT ctrl-NB-DNJ: N.S. $P = 5.9\times10^{-1}$, E| WT ctrl-PDMP: N.S. $P = 9.7\times10^{-1}$, E| WT ctrl-NB-DNJ: N.S. $P = 2.8\times10^{-1}$).

Figure S2

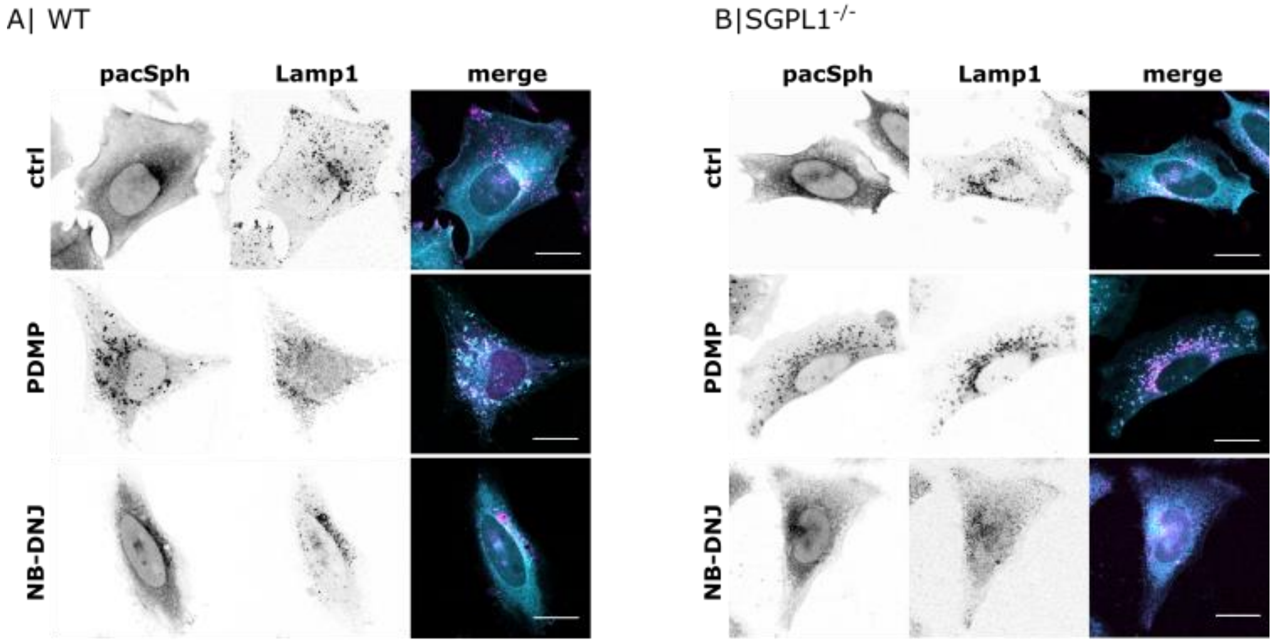

Figure S2: Confocal microscopy images of pacSph-derived lipids upon inhibitor treatment. HeLa A| WT and B| SGPL1-/- cells were treated with PDMP (20 μM) or NB-DNJ (50 μM) for 22 h and pulsed with pacSph (2 μM for 3 h). PacSph-derived lipids were cross-linked by UV-irradiation and clicked to Alexa555-azide. Co-localization with a lysosomal marker was achieved by immunofluorescence staining using Lamp1 antibody. Scale bar indicates 20 μm.

Figure S3

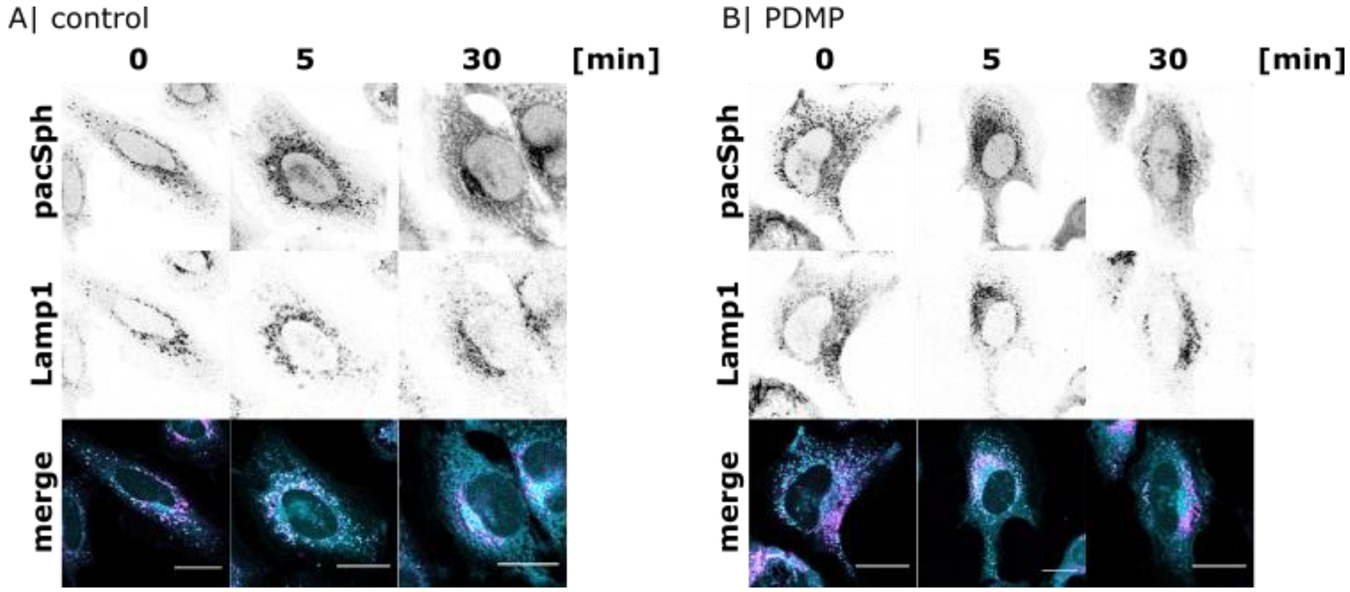

Figure S3: Confocal microscopy images of pacSph-derived lipids in short pulse-chase timpoints. HeLa SGPL1-/- A| untreated control or B| PDMP (20 μM, 22h) treated cells were pulsed for 5 min with pacSph (2 μM) and chased for 0, 5 or 30 min in lipid-free medium. Sphingolipids were cross-linked, fixed, and clicked to a fluorophore. Lysosomes were visualized by immunofluorescence staining using Lamp1 antibody. Scale bar indicates 20 μm.

Figure S4

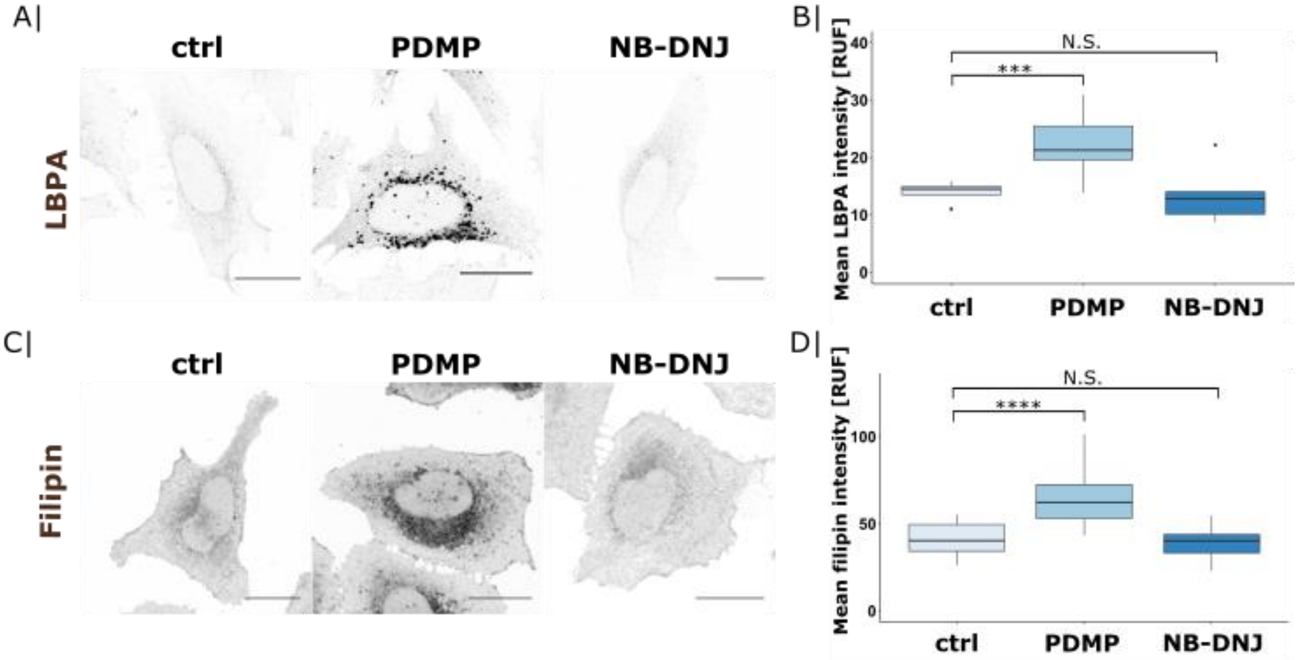

Figure S4: Immunofluorescence microscopy images of LBPA and cholesterol. HeLa WT cells treated with PDMP (20 μM, 22 h) or NB-DNJ (50 μM, 22 h) and fixed using PFA. A| LBPA was visualized using an LBPA antibody. Scale bar indicates 20 μm. B| Mean LBPA intensity was quantified (ctrl: n = 4 cells, PDMP: n = 13 cells, NB-DNJ: n = 6 cells) and presented as boxplots. Center lines show median, box limits indicate first (Q1) and third quartiles (Q3), whiskers extend to maximum distance of 1.5*IQR (interquartile range) from Q1 and Q3, respectively or to the most extreme data point

within that range. Welch two sample t-tests were performed between control and PDMP or NB-DNJ (ctrl-PDMP: *** $P = 4.4\times10^{-4}$, ctrl-NB-DNJ: N.S. $P = 7.9\times10^{-1}$). C| WT cells were treated with PDMP (20 μM, 22 h) or NB-DNJ (50 μM, 22 h), fixed and stained using 50 μg/mL filipin. Scale bar indicates 20 μm. D| Mean filipin intensity was calculated for each cell (ctrl: n = 33 cells, PDMP: n = 34 cells, NB-DNJ: n = 33 cells) and presented as boxplots. Center lines show median, box limits indicate first (Q1) and third quartiles (Q3), whiskers extend to maximum distance of 1.5*IQR (interquartile range) from Q1 and Q3, respectively or to the most extreme datapoint within that range. Welch two sample t-tests were performed between control and PDMP or NB-DNJ (ctrl-PDMP: **** $P = 1.4\times10^{-10}$, ctrl-NB-DNJ: N.S. $P = 2.7\times10^{-1}$).

Figure S5

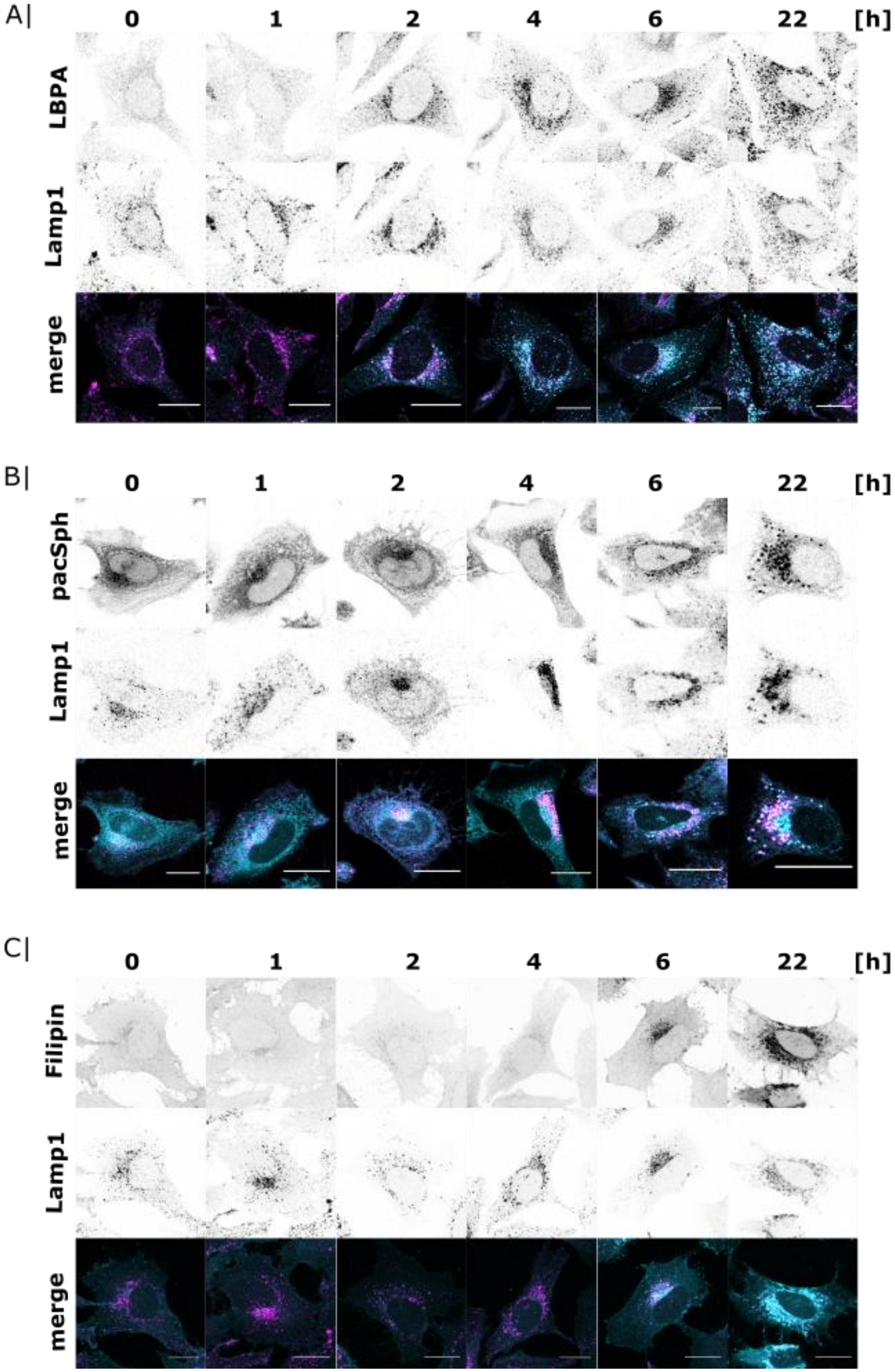

Figure S5: PDMP timecourse and its effect on lysosomal lipid accumulation. A| HeLa WT cells were treated with PDMP (20 µM) for 0 - 22 h, fixed with PFA and stained using LBPA and Lamp1 antibodies, respectively. B| Confocal microscopy images

of sphingolipids and lysosomes. HeLa SGPL1$^{-/-}$ cells were treated with PDMP (20 µM for 0 – 22 h), pulsed with pacSph (2 µM for 5 min) and afterwards chased for 15 min in medium without lipids. Subsequently sphingolipids were crosslinked by UV-irradiation and cells were methanol fixed. Crosslinked pacSph was clicked to Alexa555-azide and lysosomes were visualized using immunofluorescence staining against Lamp1. C| Confocal microscopy images of HeLa WT cells treated with PDMP (20 µM for 0 – 22 h), PFA fixed and stained with 50 µg/mL filipin followed by immunofluorescence staining using Lamp1 antibody. Scale bar indicates 20 µm.

Figure S6

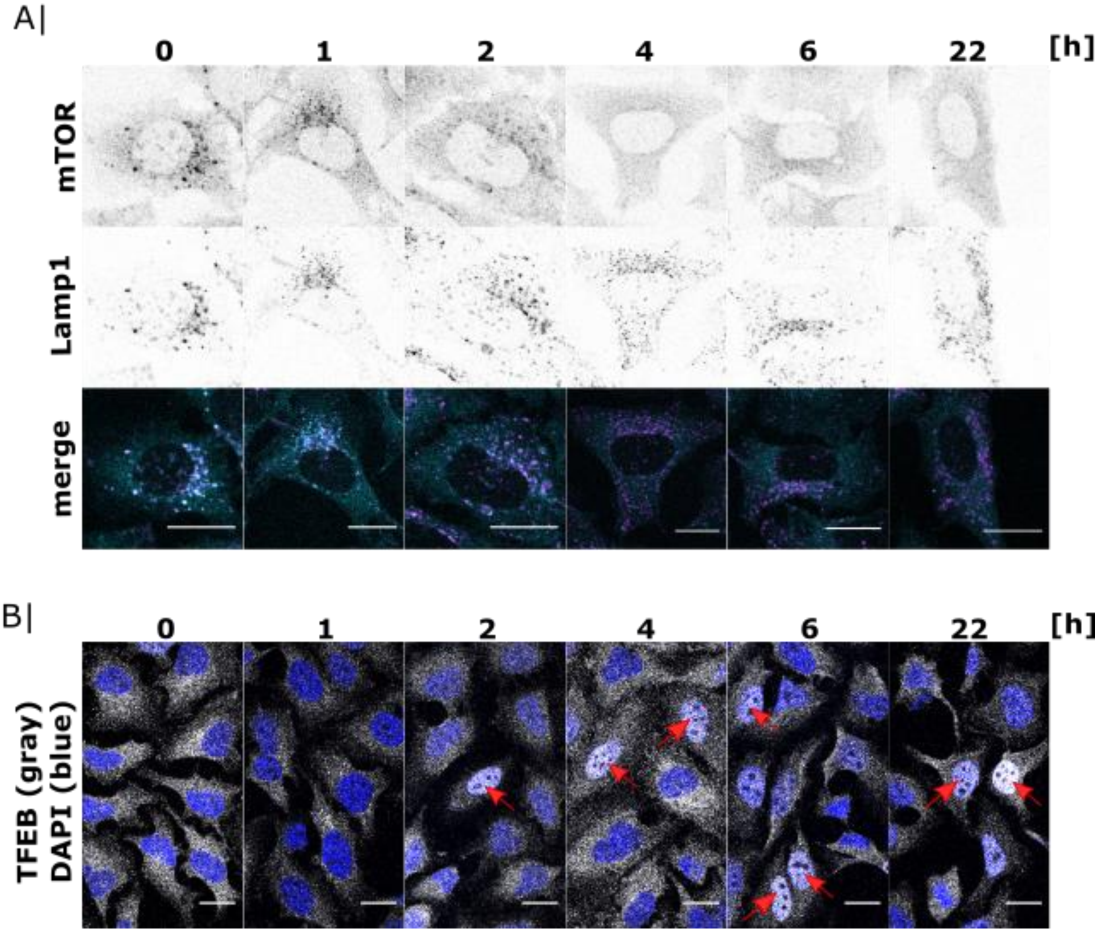

Figure S6: PDMP time-course and its effect on mTOR and TFEB translocation. A| HeLa WT cells were treated with PDMP (20 µM) for 0 - 22 h, fixed with PFA and stained using a mTOR-antibody. Co-localization with lysosomes was achieved by Lamp1 antibody staining. B| HeLa WT cells were treated with PDMP (20 µM) for 0 - 22 h, fixed with PFA and stained using a TFEB antibody (gray). For colocalization with the nucleus, cells were stained with DAPI (blue). Red arrows illustrate cells with nuclear localization of TFEB. Scale bar indicates 20 µm.
